# Link Clustering Reveals Structural Characteristics and Biological Contexts in Signed Molecular Networks

**DOI:** 10.1371/journal.pone.0067089

**Published:** 2013-06-24

**Authors:** Chen-Ching Lin, Chia-Hsien Lee, Chiou-Shann Fuh, Hsueh-Fen Juan, Hsuan-Cheng Huang

**Affiliations:** 1 Institute of Biomedical Informatics, Center for Systems and Synthetic Biology, National Yang-Ming University, Taipei, Taiwan; 2 Graduate Institute of Biomedical Electronics and Bioinformatics, National Taiwan University, Taipei, Taiwan; 3 Department of Life Science, Institute of Molecular and Cellular Biology, Center for Systems Biology, National Taiwan University, Taipei, Taiwan; Tel Aviv University, Israel

## Abstract

Many biological networks are signed molecular networks which consist of positive and negative links. To reveal the distinct features between links with different signs, we proposed signed link-clustering coefficients that assess the similarity of inter-action profiles between linked molecules. We found that positive links tended to cluster together, while negative links usually behaved like bridges between positive clusters. Positive links with higher adhesiveness tended to share protein domains, be associated with protein-protein interactions and make intra-connections within protein complexes. Negative links that were more bridge-like tended to make interconnections between protein complexes. Utilizing the proposed measures to group positive links, we observed hierarchical modules that could be well characterized by functional annotations or known protein complexes. Our results imply that the proposed sign-specific measures can help reveal the network structural characteristics and the embedded biological contexts of signed links, as well as the functional organization of signed molecular networks.

## Introduction

Biological processes in living cells are usually accomplished by numerous interactions between biological molecules (genes, proteins and other cell components) at various scales. Therefore, molecular networks, which are comprised of biological molecules and interactions between them, can provide a comprehensive interpretation of complicated biological systems in living cells and have become a key approach to understanding biological systems [1]–[4]. Investigation of the network structure has been used to reveal biological contexts embedded in molecular and cellular networks [5]–[7]. For example, Lin *et al*. studied the complete graphs in protein-protein interaction networks, and identified the essential cores in protein networks of *Escherichia coli* and *Saccharomyces cerevisiae* [5]; Roth *et al*. used the minimum spanning trees to extract the most relevant information contained in the gene network of *Bacillus subtilis* [6]; Madi *et al*. also analyzed the minimum spanning trees in immune networks, and found different conservative level between mothers’ and newborns’ networks [7]. Link clustering denotes the overlap between neighboring links and has been used to identify communities in molecular and social networks [8], [9]. Essentiality of a protein in the interaction network was found to be highly associated with the link clustering level of the interactions connecting it [10]. Moreover, Solava *et al*. utilized link clustering to predict new pathogen-interacting proteins which possibly play the role of drug target candidates [11].

Many molecular networks, such as the genetic interaction network (GIN) and the gene coexpression network (CEN), are signed undirected networks that consist of positive and negative links (genetic interactions or gene coexpression). Genetic interactions (GIs) describe that double mutants confer a significant deviation of phenotype from the expected value [12]. This expected value of phenotype change is referred to as the combination effect of two single mutations [13]. Positive GIs are when the phenotypic changes of double mutants are equivalent to or less severe than expected, such as synthetic suppression or rescue. In contrast, negative GIs are when double mutants display a more severe phenotype than expected, such as synthetic lethality or sickness [14], [15]. Genes with positive GIs have been referred to as alleviating or epistatic interactions, while those with negative GIs are usually thought to participate in parallel biological pathways. Thus, single mutants are compatible with continued viability, while the double mutants damage viability [14]. Previous studies have reported that genes with similar patterns of GI profiles tended to participate in the same biological pathways or processes [16], [17]. Gene coexpressions (CEs) are measured by expression correlations between genes, usually measured by the Pearson correlation coefficient (PCC) or other metrics. The CEN collates correlated genes under well-designed experimental states. In CENs, simultaneously expressed gene-pairs form positive CEs, while inversely expressed pairs form negative CEs.

Herein, considering the essential differences between positive and negative links, we proposed four measures of link-clustering coefficients (LCs), which were used to evaluate the proportions of common interacting partners, also called neighbors, between linked molecules. By applying LCs to study the network structure of a CEN, we found that positive links were more adhesive and tended to cluster together, while negative links were more dispersive and usually behaved like bridges between positive clusters. Interestingly, a similar network structure was also observed in the GIN. Additionally, the proposed LC could be further used to reveal hidden biological contexts of signed links and to uncover the network modules that are well characterized by functional annotations or known protein complexes.

## Results

### Coexpression Network (CEN)

#### Network structure of the CEN

**Figure 1:**
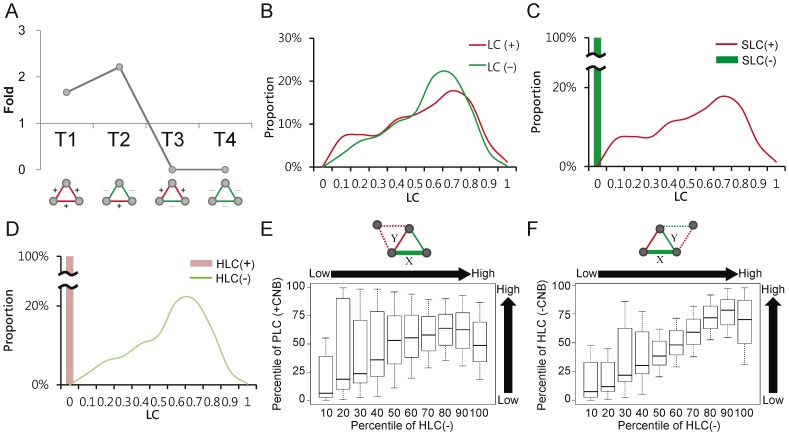
Structural properties of the CEN. (A) Frequency of signed triads in CEN. According to combinatorial patterns of signed links, four types of triads are listed. Fold is the ratio of observed number of triads to the average number of random triads. (B)–(D) LC, SLC, and HLC distributions of positive/negative links in the CEN. The values shown on the x-axis are the upper bounds of the corresponding LC intervals. (E) Median of PLC of positive CEs linking to the common neighbor (CNB) that contributed to the HLC of the observed negative CE with increasing HLC(–). The Pearson’s correlation between PLC of positive CEs linking to the common neighbor and HLC(–) is 0.52 (*P*<2.2×10^−16^). (F) Median of HLC of negative CEs linking to the common neighbor (CNB) that contributed to the HLC of the observed negative CE with increasing HLC(–). The Pearson’s correlation between HLC of negative CEs linking to the common neighbor and HLC(–) is 0.64 (*P*<2.2×10^−16^).

Coexpression networks consist of gene pairs with similar or opposite gene expression profiles. Here, we defined coexpression as a positive link and anti-coexpression as a negative link, following the sign of the correlation coefficient between expression profiles. Since correlations had transmission characteristics (Figure S1), two genes with common coexpressed and/or anti-coexpressed genes in the CEN are expected to express simultaneously. It could lead CEN to possess specific network structural properties, such as distribution of triads – the smallest units of the complete graph. There are four possible types of triads according to the combinatorial patterns of the three interconnected signed links, denoted T_1_–T_4_ in Figure 1A. The frequencies of each type of triad were assessed by the ratio of the observed number for each triad-type to the corresponding expected value from random shuffling of the signs of links (more details in Text S1). As expected, we observed that T_1_ (+++) and T_2_ (+−−) were significantly over-represented, while T_3_ (−++) and T_4_ (−−−) were totally absent (Figure 1A and Table S1). In other words, positive CEs tend to cluster with co-positive or co-negative CE neighbors, while negative CEs tend to cluster with hybrid ones. This observation suggested that positive and negative CEs should have distinct clustering features. Thus, we applied an LC that measured the proportion of common neighbors between two linked nodes to assess the aggregation characteristics of links [9]. We first disregarded the signs of the interconnected links by the conventional LC definition, and found that the LC distribution of negative CEs was similar to that of positive CEs (Figure 1B). It suggested that both types of CEs could cluster with other CEs, but the difference between the clustering properties of positive and negative CEs were indistinguishable using unsigned LCs. To differentiate the clustering characteristics of positive and negative links, we took the signs of clustering links into consideration, dividing unsigned LCs into two sign-specific groups: Same (SLC), which considers only the neighboring links of the same signs and Hybrid (HLC), which considers neighboring links of opposite signs. We found that SLC of positive links, SLC(+), remained similar to unsigned LC(+) while that of negative links, SLC(−), were all zero (Figure 1C). On the other hand, HLC of negative links, HLC(−), remained similar to unsigned LC(−) while HLC(+) were all zero (Figure 1D). Apparently, clustering properties of positive and negative CEs can be distinguished by our proposed sign-specific LCs. According to the signs of paired links connecting to their common neighbors, SLC can be further divided into two subtypes, PLC (LCs with two positive signs) and NLC (LCs with two negative signs). Both of their distributions for positive links were similar to SLC(+) (Figure S2a). These results were consistent with the expected network structural characteristics of CEN. Additionally, we found that negative CEs with higher HLC tended to recruit common neighbors with higher PLC(+) and HLC(−) (Figure 1E, F). This suggested that positive CEs linking to the common neighbors that contributed to HLC(−) tended to form positive clusters and negative ones tended to connect to (other) positive cluster(s). In other words, we can infer that positive links are more adhesive and tend to cluster together while negative links are more dispersive and usually behave like bridges between positive clusters. Altogether, above results suggested that the proposed signed LC was capable of reflecting and even highlighting the structural characteristics of CEN.

#### Biological contexts in the CEN

**Figure 2:**
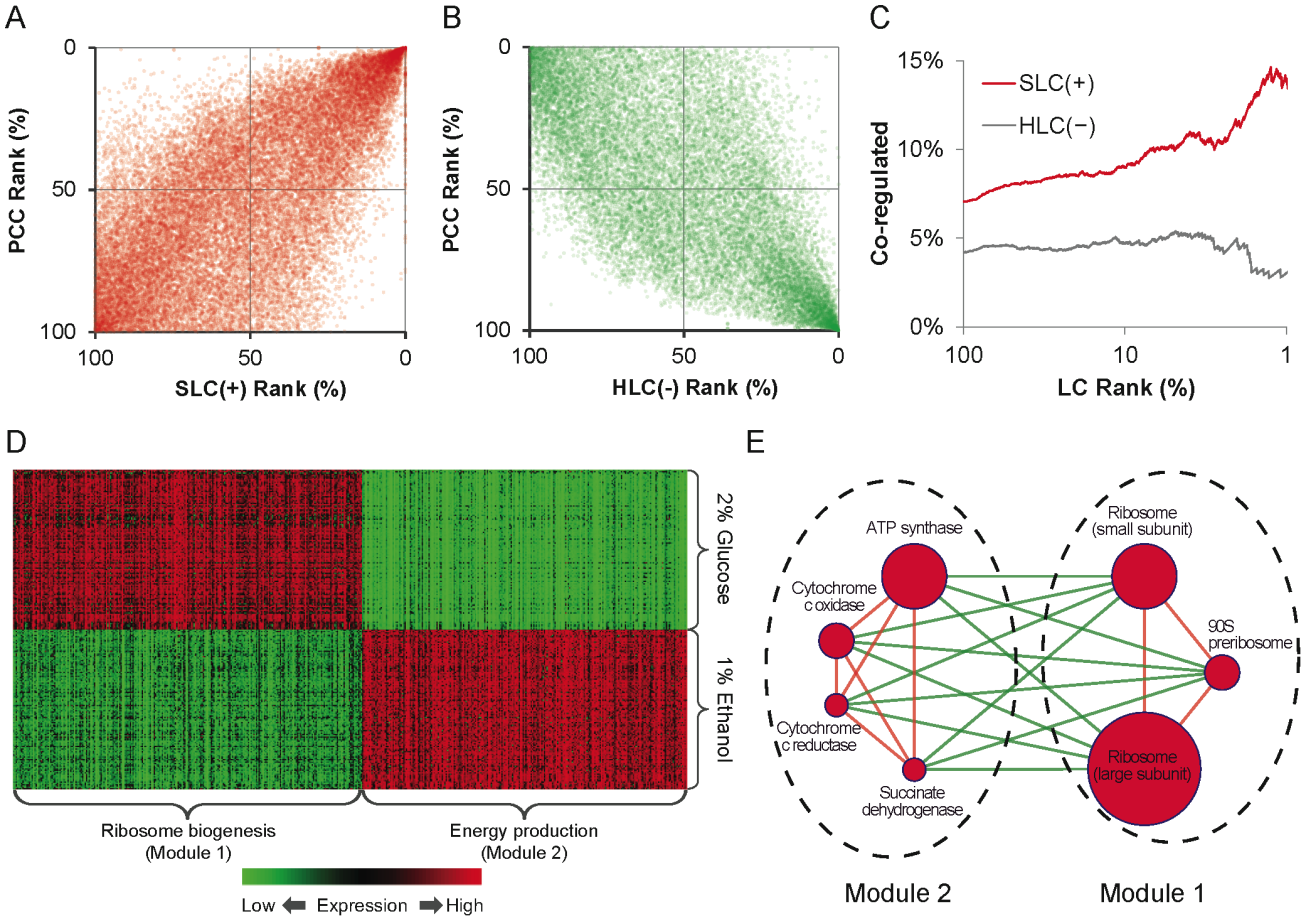
Biological contexts embedded in the CEN. Rank 0% and 100% represent the highest and lowest value of corresponding measurement, respectively. (A)(B) Two positive (negative) CE genes with higher PLC (HLC) tended to coexpress (anti-coexpress) with each other more. (C) Two positive CE genes with higher PLC or NLC tended to be regulated by the same transcription factors. (D) Expression profiles of the two largest functional modules. (E) Well-known protein complex inside selected two largest modules. Node size represents the number of genes covered by the corresponding sub-module. Node color represents the density of positive CEs involved in the sub-module. Red (green) links indicate that CEs between two sub-modules are all positive (negative).

The CEN was constructed by discretizing the correlations between expression profiles of gene pairs. Each individual link in the CEN preserved only the binary information of whether the two linked genes were coexpressed (for those with a significant positive correlation coefficient above a certain threshold) or anti-coexpressed (with a significant negative correlation coefficient). Although such a network representation seemingly diminishes the quantitative information of individual links, the quantitative correlation information was, in fact, embedded in the network structure and could be recovered to a certain extent. A pair of genes with highly correlated expression profiles was expected to share a larger amount of commonly linked genes, resulting in a higher SLC, as well as PLC and NLC. On the other hand, those with highly anti-correlated expression profiles were expected to share genes with opposite types of links, resulting in a higher HLC. Indeed, we observed a strong correlation between SLC and PCC for gene pairs with positive links (Figure 2A; see also Figure S2b for similar characteristics of PLC and NLC), and a strong anti-correlation between HLC and PCC for negative links (Figure 2B). Furthermore, the proportion of coregulated gene pairs increased along with SLC for positive links, but not for HLC of negative links (Figure 2C; see also Figure S2c for similar characteristics of PLC and NLC). In other words, the coexpressed gene pairs sharing more common coexpressed or anti-coexpressed partners tended to be regulated by the same transcription factors. Therefore, it suggested that sign-specific LCs could reveal the embedded quantitative magnitude of coexpression, as well as the biological contexts involved in the CEN.

Next, we applied the predefined similarity measure, which was derived from the summation of two same-sign LC subtypes, PLC and NLC, to cluster positive links for identification of potential functional modules (see Materials and Methods). Among 34 identified modules (size ≥3), we focused on the two largest modules, which covered 263 and 245 genes, respectively. Links inside these two modules are all positive, but those between them are all negative, which is consistent with the observed structural characteristics of the CEN. Positive links inside these two modules tended to have higher PLC and NLC (≥0.5, Figure S2d,e), while negative links between modules tended to have a higher HLC (≥0.5, Figure S2f). Again, positive CEs with high PLC or NLC are modular, while negative CEs with a high HLC are bridge-like. The gene expression profiles of these two modules displayed similar patterns inside modules, but were opposite to each other between modules under different conditions of nutrition sources, i.e., 1% ethanol and 2% glucose (Figure 2D). Notably, we chose these two modules only according to the proposed signed LC and their size. The enriched biological functions of these two modules were ribosome biogenesis and energy-production-related functions, respectively (Table S2 and S3). We noted that the large and small subunits of ribosome and 90S preribosome were involved in the largest module and that ATP synthase, cytochrome c oxidase, cytochrome c reductase and succinate dehydrogenase were involved in the second largest module (Figure 2E and Figure S2g). These well-known protein complexes are directly associated with ribosome biogenesis or energy production. Additionally, we observed that the largest module was activated by 2% glucose and repressed by 1% ethanol, in contrast to the second largest module, which behaved in the opposite manner (Figure 2D). It was reported that glucose can transcriptionally repress TCA cycle genes, decrease respiratory activity and activate ribosome protein genes as sufficient amounts of glucose are available to support cell growth [18]. On the other hand, under ethanol stress, yeast initially struggles to maintain energy production by increasing expression of genes associated with energy-generating activities and decreasing expression-rates of genes associated with energy-demanding processes, such as growth [19]. In summary, these results indicate that the proposed LC has potential to reveal the biological contexts of signed links and the functional modules, such as protein complexes, in signed molecular networks.

### Genetic Interaction Network (GIN)

#### Network structure of the GIN

**Figure 3:**
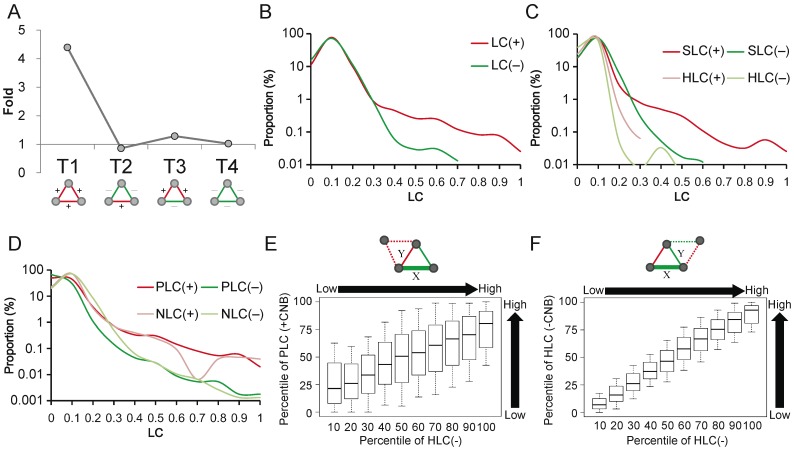
Structural properties of the GIN. (A) Frequency of signed triads in GIN. (B)–(D) LC, SLC, HLC, PLC, and NLC distributions of positive/negative links in GIN. (E) Pearson’s correlation between PLC of positive GIs linking to the common neighbor (CNB) and HLC(–) is 0.57 (*P*<2.2×10^−16^). (F) Pearson’s correlation between HLC of negative GIs linking to the common neighbor (CNB) and HLC(–) is 0.88 (*P*<2.2×10^−16^).

Unlike CEs, GIs didn’t possess transitive property. In the GIN of *Saccharomyces cerevisiae*, we observed that four types of triads were present and only T_1_ (+++) was significantly over-represented (Figure 3A and Table S1). This resembles the characteristics of CEN–i.e., that positive links tend to cluster with positive link neighbors–although the triads involving negative links behaved differently in the GIN. The unsigned LC distributions of positive GIs also showed a higher tail than negative ones (Figure 3B). To resolve what kind of aggregation forms positive GIs prefer, we analyzed the signed LC of GIs. SLC(+) distributed toward higher coefficients than the other three types of signed LC, HLC(+), SLC(−), and HLC(−) (Figure 3C). Additionally, PLC(+) and NLC(+) also distributed with higher tails than PLC(−) and NLC(−), respectively (Figure 3D). These results suggest that the following: (1) positive GIs tend to form clusters with co-positive or co-negative GI neighbors rather than with hybrid GI neighbors; (2) compared with positive GIs, negative GIs disfavor clustering. To clarify the characteristics of negative links in GIN, we interrogated the clustering characteristics of the hybrid links contributing to HLC(−). We found that positive GIs of hybrid links involved in HLC(−) tended to form positive clusters (Figure 3E). Furthermore, HLC of negative GIs in HLC(−) hybrid links positively correlated with the observed HLC(−) (Figure 3F). These findings suggested that the negative GIs with high HLC tended to act as bridges between positive clusters. Although the triad and LC distributions of the GIN differ from the CEN, they share similar features, i.e., that positive GIs tend to cluster together and negative GIs usually behave like bridges between positive clusters.

#### Biological contexts embedded in genetic interaction links

**Figure 4:**
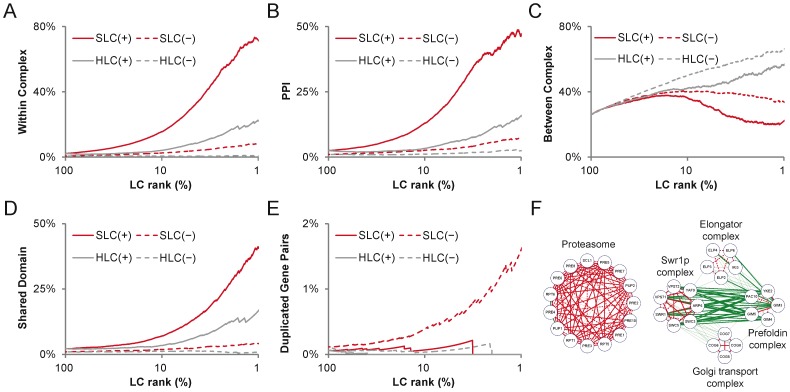
Biological contexts embedded in GIs. (A)–(E) The correlations between GI and biological contexts. The x-axis represents the percentage of ranked LC value, and the value 1 means the top 1% highest LC value. The y-axis represents accumulated proportions of GIs with corresponding biological contexts. (F) Example complexes formed by positive GIs with the top 1% highest SLC. Positive and negative GIs are represented by red and green links, respectively. Dashed links are PPIs. Bold links are GIs with the top 1% highest SLC or HLC, and thin ones are the other GIs.

Genetic interactions are measured by the phenotypic change of perturbed living cells, and hence are thought to make functional connections within and/or between biological processes [3], [17], [20]–[22]. Previous studies have reported that positive GIs tend to appear between gene pairs with protein-protein interactions (PPIs) or participate in the same protein complex, while negative GIs tend to be interconnections between different protein complexes [23]–[27]. Herein, we observed that positive GIs with higher SLCs tended to be PPIs or intra-connections within the same protein complex (Figure 4A, B), and negative ones with higher HLCs tended to be interconnections between different protein complexes (Figure 4C). We also found that proteins encoded by genes that formed positive GIs with higher SLCs tended to share the same protein domains (Figure 4D). These observations suggest that positive GIs with higher SLCs could imply a stronger functional relationship or homogeneity between genetic interacting genes. SLC(+) and HLC(−) not only reflect the network topological properties–i.e., the intramodularity of positive GIs and bridgeness of negative GIs–but also help reveal the biomolecular complex structure and organization involved. For example, we found that several protein complexes were enriched by the positive GIs with the top 1% highest SLC (Figure 4F; *p*<<0.0001, Fisher’s exact test); 96% of the negative GIs among these complex subunit genes made interconnections between different complexes and were enriched in the top 1% highest HLC (*p*<<0.0001, Fisher’s exact test). Notably, all the PPIs among them were from positive GIs with the highest 1% SLC and intra-connections within the complex. Their shared protein domains were mostly related to proteasome subunits and prefoldin. On the other hand, previous studies have reported that negative GIs possibly reflect the evolutionary relationship between two genetic interacting genes [28]–[31]. Interestingly, we found that only gene pairs of negative GI with higher SLCs tended to be duplicated genes (Figure 4E). Since higher SLC implies potentially higher functional homogeneity, this observation might result from the functional compensatory relationship between negative genetic interacting genes [31].

### Genetic Interaction Modules

**Figure 5:**
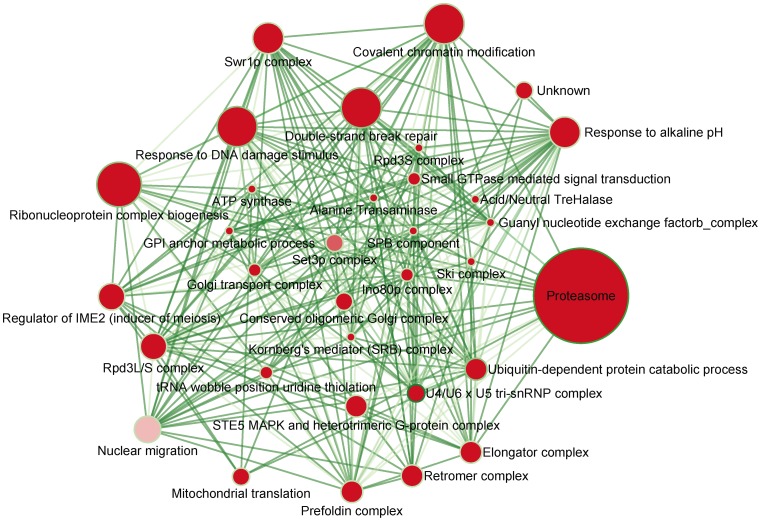
Map of genetic interaction modules. Each node represents a module clustered by positive GIs, and each edge represents a bunch of negative GIs between different modules. Node size indicates the number of genes in each module and node color intensity indicates the density of positive GI. Edge width indicates the number of GIs between modules and edge color intensity indicates the proportion of negative GI. Color intensity of node border indicates the density of negative GI in each module.

After investigating the structure and biological contexts of GIN, we noticed that positive GIs tended to form functionally homogeneous modules. To discover these modules inside the GIN, we applied single-linkage hierarchical clustering with the LC-based similarity score of positive GIs, ranked in descending order, and utilized partition density [8] to determine the similarity score cut-off of the optimal modular structure. As the cut-off of positive GIs increased, partition density was first elevated to a maximal value and then decreased (Figure S3a), implying that the positive GIN did contain a local modular structure. However, partition density was only decreased when the similarity score cut-off of negative GIs increased (Figure S3b), which implied that the negative GIN consisted of no local denser subnetworks. When the similarity score of positive GI that corresponded to the maximal partition density was applied, 33 positive modules that consisted of more than three genes were discovered (Figure 5). Indeed, over 90% of modules possessed highly intraconnected positive GIs (positive density ≥0.5) and almost 80% of them contained no negative GI (Figure S3c). 70% of link sets between modules only contained negative GIs (Figure S3d). Additionally, these modules could be well characterized by known protein complexes or biological processes (Figure 5). Some of the modules, such as “response to DNA damage stimulus” and “double-strand break repair”, have been reported to be synthetic lethal with each other [30]. More importantly, this implies that the gene-based GIN can be summarized as a module-based network by applying the LC-based similarity score to cluster positive GIs.

## Discussion

In this study, we applied a rigorous threshold to define the coexpression links in a CEN, |PCC| ≥0.9. Because of the transmission property of correlation, only type 1 and type 2 triads are allowed, while type 3 and type 4 are not.In GIN, all four types of triads were observed, which might imply that the GIN possessed a triad-enriched network structure. On the other hand, GIs measure the phenotypic relevance between genes and the changes of phenotypes often relate to complicated and numerous biological processes. Consequently, GIs are usually thought to be subtle and to underlie diverse biological contexts. Therefore, triple GIs that formed triads in GIN might easily be derived from different biological contexts, and thus they might not follow the transitory information.

According to the *structural balance theory* – proposed by Heider in the 1940s [32] and formulated by Cartwright and Harary in graph theory [33], type 1 and type 2 triads are balanced and type 3 and type 4 are unbalanced. Therefore, CEN is structurally balanced and follows the two structure theorems [33], [34] summarized by Hummon and Doreian [35]: *A network is balanced if and only if the network can be divided into two or more subnetworks, wherein links in the same subnetwork are all positive and between different subnetworks are negative*. In the GIN, four types of triads were present, while only type 1 was significantly over-represented (Figure 1A and Table S1). This suggests that the GIN was weakly structurally balanced [34] and, thus, abates the requirement of T_2_ over-representation. In summary, the signed molecular network is (weakly) structurally balanced and T_1_ (three mutually positively linked genes) is significantly over-represented relative to chance.

In the proposed module map, one notable interaction is between “double-strand break repair” and “Swr1p complex”. In the double-strand break repair module, XRS2 and RAD50 are parts of the MRE11-RAD50-XRS2 (or MRX) complex, which plays a vital role in both homologous recombination (HR) repair and non-homologous end-joining (NHEJ) repair [36]. Additionally, RAD51, RAD52, RAD54, and RAD55 participate in the primary repair process [37]. The TOP3-RMI1-SGS1 complex is required to resolve the DNA intermediate structure, which is produced in the final steps of HR [38]. Genes in the “Swr1p complex” module are part of the histone post-modification pathway [39]. In this pathway, H2BK123 is ubiquitinated by the Rad6-Bre1 complex [40]. The ubiquitination requires the presence of the Paf1 complex, which contains two subunits, RTF1 and CDC73, in this module [39]. After the ubiquitination of H2BK123, H3K4 is trimethylated by the Set1 complex, which contains four subunits, SWD1, SWD3, SDC1 and BRE2, in this module [41]. The H3K4 trimethylation is related to the NHEJ repair pathway [40]. In agreement with the balance structure of the signed molecular network, the density of positive GIs in these two modules are 0.5 and 0.7, respectively, and links between these two modules are almost completely negative (96%). As described above, genes in the double-strand break repair module are part of the HR repair pathway, and genes in the Swr1p complex module participate in NHEJ-related histone post-translational modifications. HR and NHEJ are two major DNA double-strand repair pathways of the yeast cell [42]. This suggests that these two modules participate in two different pathways with the same or similar output and, therefore, they should be able to complement each other.

In this study, we applied the signed LC to study the network structure of the signed molecular network and successfully revealed the differences of clustering characteristics between positive and negative links. The results showed that positive links tend to cluster together, while negative links are more dispersive and usually make interconnections between positive clusters. Furthermore, the signed LC facilitated the discovery of the diverse biological contexts covered by signed links and the functional modules within signed molecular networks.

## Materials and Methods

### Coexpression and Genetic Interaction Networks

To construct the CEN, we downloaded the expression profiles of yeast genes from Gene Expression Omnibus (GEO), accession number GSE9376 [43], containing 6,253 genes and 246 samples in various nutrition sources. The correlations between genes were evaluated by the Pearson correlation coefficient (PCC). To stress the correlations between genes, paired genes with PCC ≥0.9 were defined as positive coexpression and those with PCC ≤ −0.9 as negative. The studied CEN consisted of 1,240 genes and 48,497 coexpression links (28,651 positive and 19,846 negative).

The yeast genetic interactions were downloaded from BioGRID 3.1.72 [44]. We retrieved “synthetic rescue” and “positive genetic” relationships between genes as positive GIs, and “synthetic lethality” and “negative genetic” ones as negative GIs. In addition, 448 ambiguous GIs were removed from this dataset. After this filtration, 5,084 genes and 91,743 GIs (15,821 positive and 75,922 negative) were included in the yeast GIN.

We applied the algorithm proposed by Lin *et al.* [5] to identify and count the number of triads in the CEN and GIN.

### Link-clustering Coefficient

Given a network composed of nodes and links connecting paired nodes (edges), the link-clustering coefficient (LC) measures the proportions of shared neighbors (common linking partners) between linked molecular pairs, and is defined as:
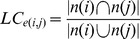
where *LC_e(i,j)_* is the LC of the link *e* formed by node *i* and *j*. Note that *n(i)* (*n(j)*) is the excess neighbors of node *i* (*j*) excluding node *j* (*i*). Previous studies have noted that biological molecules would be likely to share similar functions with their neighbors [45], [46]. Thus, a higher LC means a larger proportion of shared neighbors and implies higher functional similarity between two interacting molecules. Herein, LC was calculated for positive and negative links in signed molecular network separately. LC(+) and LC(−) denoted the LCs of positive links and the LCs of negative links, respectively. Further, according to the signs of paired links connecting to the common neighbors, LC can be classified into two subtypes, same (SLC, +/+ or −/−) and hybrid (HLC, −/+ or +/−), which are defined as:



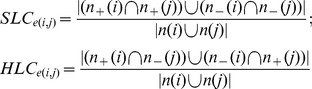
where 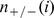 (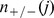) is the excess positive/negative neighbors of node *i* (*j*) excluding node *j* (*i*). Based on its definition, SLC can be further categorized into two subtypes, positive (PLC) and negative (NLC), which are defined as:



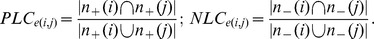



### Revealing Biological Contexts and Communities in Signed Networks

Herein, several biological relationships between genes–PPI, within/between protein complex, shared protein domain and duplicated genes–were used to discover the embedded biological contexts of GIs (more details in Text S1) [47]–[51]. The proportions of biological contexts covered by positive/negative GIs were calculated and referred to as the relevance of biological contexts to GIs.

To discover the biological communities, single-linkage hierarchical clustering was applied with the similarity score defined as:
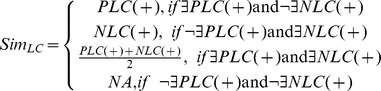



The threshold for cutting this dendrogram to yield communities was determined by maximum partition density, which was introduced by Ahn *et al.* [8]. The potential biological processes of each community were investigated by functional enrichment analysis (more details in Text S1).

## Supporting Information

Figure S1 **Correlation transmission.** Correlation transmission via common (a) co-expressed and/or (b) anti-expressed neighbors.
(TIF)

Figure S2 **Biological context revealed by LC of CE.** (a) PLC and NLC distributions of positive/negative links in CEN. The values shown on the x-axis are the upper bounds of the corresponding LC intervals. (b) Two positive CE genes with higher PLC or NLC tended to have higher rates of coexpression with each other. Red points: PLC(+); Green points: NLC(+). (c) Two coexpressed genes that shared more common coexpressed (PLC) or anti-expressed (NLC) partners tended to be regulated by the same transcription factors. (d) – (f) LC distributions of the two largest modules. (g) Coexpression subnetworks of seven well-known protein complexes involved in the two largest modules.
(TIF)

Figure S3 **LC-score vs. partition density of GIN and GI density of discovered modules.** (a) LC-score vs. partition density of positive GIN. (b) LC-score vs. partition density of negative GIN. (c) Distributions of positive/negative GI density of discovered modules. (d) Distributions of negative GI proportion of meta-links between modules.
(TIF)

Table S1 **Number of triads in CEN and GIN.**
(PDF)

Table S2 **The top twenty enriched functions of the largest module in the CEN.**
(PDF)

Table S3 **The top twenty enriched functions of the 2^nd^ largest module in the CEN.**
(PDF)

Text S1 **Supplementary methods.**
(PDF)
