# Supplementary figures and images for: Link Clustering Reveals Structural Characteristics and Biological Contexts in Signed Molecular Networks
Source: PLoS One. 2013 Jun 24;8(6):e67089. doi: 10.1371/journal.pone.0067089 (PMC3691148; doi:10.1371/journal.pone.0067089)

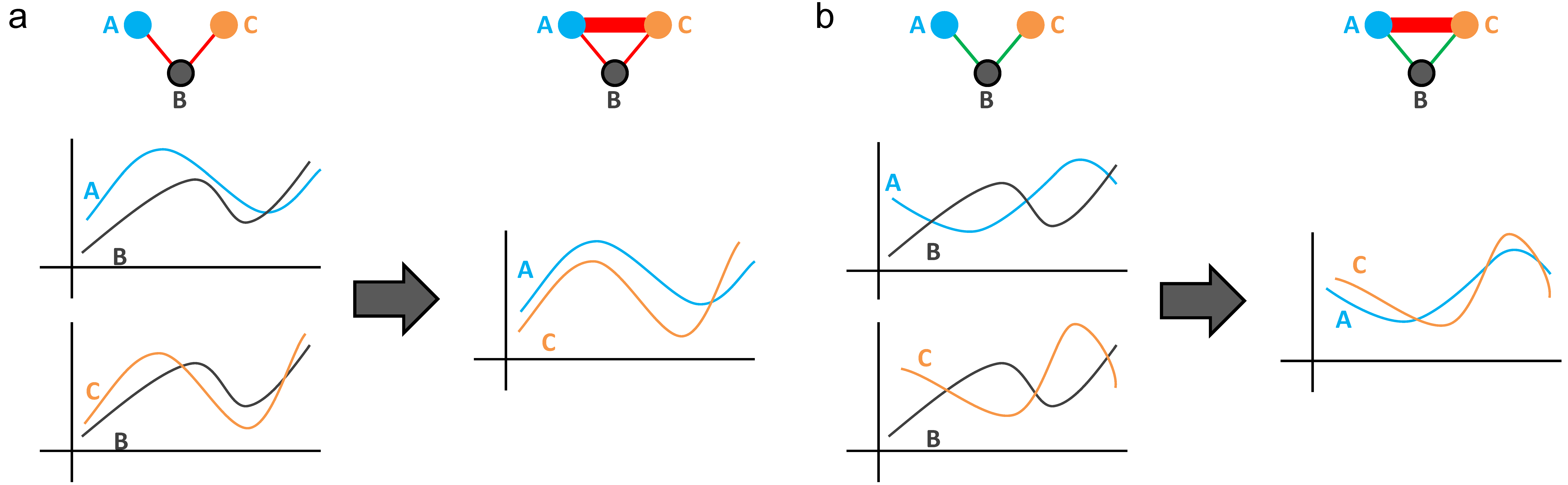

Supplement: Figure S1 — Correlation transmission. Correlation transmission via common (a) co-expressed and/or (b) anti-expressed neighbors. (TIF) [file pone.0067089.s001.tif]

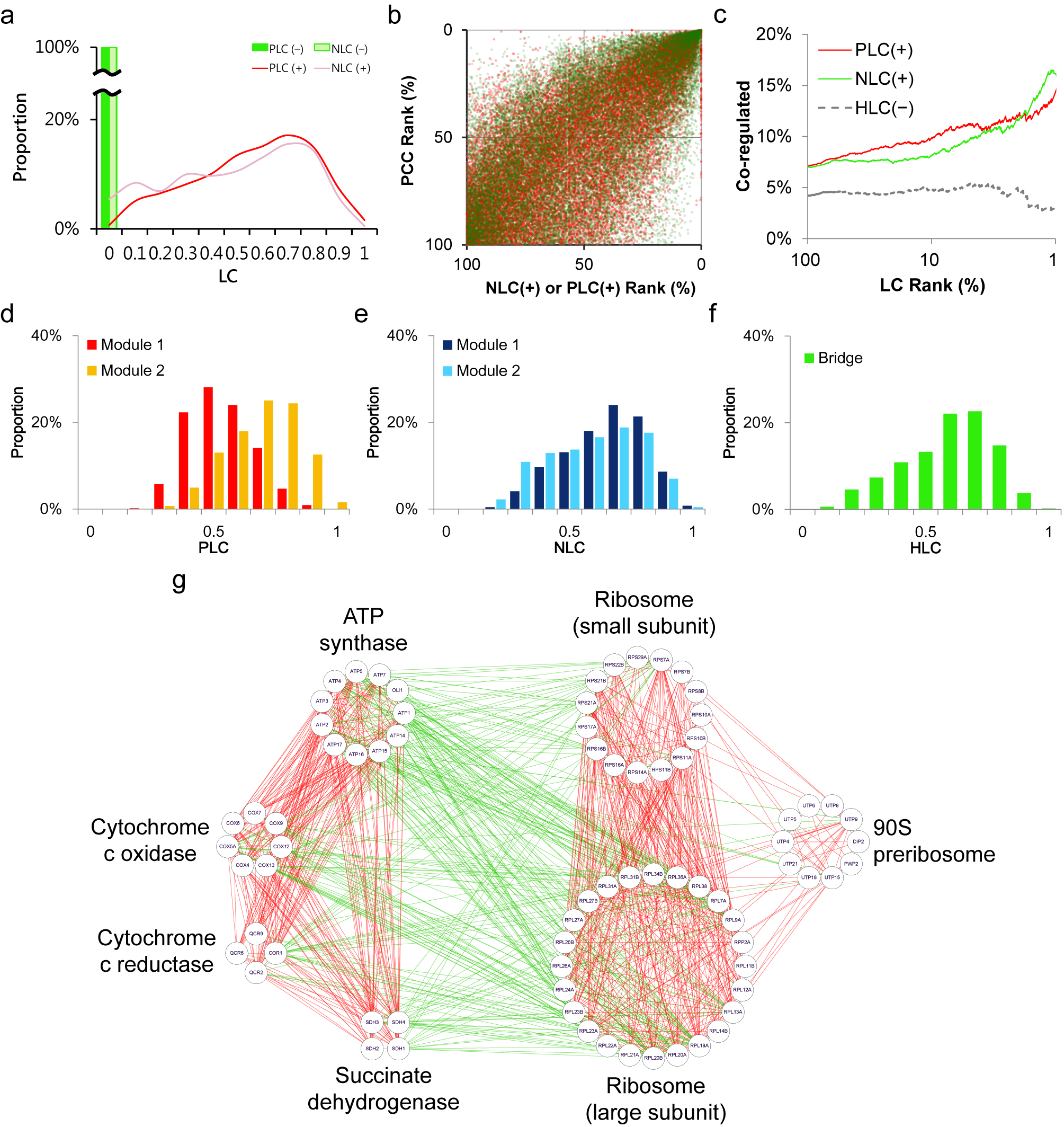

Supplement: Figure S2 — Biological context revealed by LC of CE. (a) PLC and NLC distributions of positive/negative links in CEN. The values shown on the x-axis are the upper bounds of the corresponding LC intervals. (b) Two positive CE genes with higher PLC or NLC tended to have higher rates of coexpression with each other. Red points: PLC(+); Green points: NLC(+). (c) Two coexpressed genes that shared more common coexpressed (PLC) or anti-expressed (NLC) partners tended to be regulated by the same transcription factors. (d) – (f) LC distributions of the two largest modules. (g) Coexpression subnetworks of seven well-known protein complexes involved in the two largest modules. (TIF) [file pone.0067089.s002.tif]

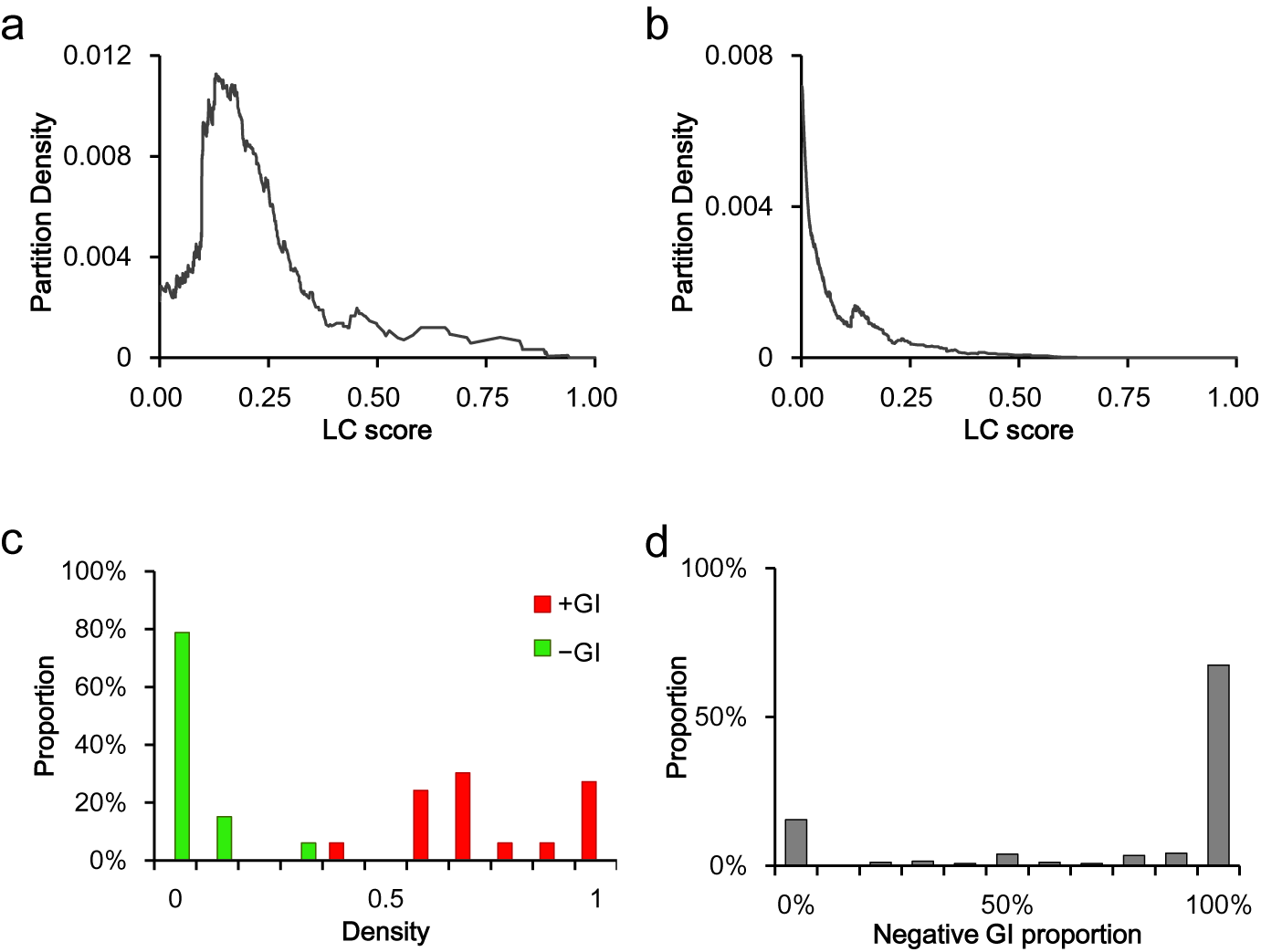

Supplement: Figure S3 — LC-score vs. partition density of GIN and GI density of discovered modules. (a) LC-score vs. partition density of positive GIN. (b) LC-score vs. partition density of negative GIN. (c) Distributions of positive/negative GI density of discovered modules. (d) Distributions of negative GI proportion of meta-links between modules. (TIF) [file pone.0067089.s003.tif]
