# Supplementary material for: Link Clustering Reveals Structural Characteristics and Biological Contexts in Signed Molecular Networks
Source: PLoS One. 2013 Jun 24;8(6):e67089. doi: 10.1371/journal.pone.0067089 (PMC3691148; doi:10.1371/journal.pone.0067089)
Supplement: Table S1 — Number of triads in CEN and GIN. (PDF) [file pone.0067089.s004.pdf]

Link clustering reveals structural characteristics and biological contexts in signed molecular networks

Chen-Ching Lin, Chia-Hsien Lee, Chiou-Shann Fuh, Hsueh-Fen Juan, Hsuan-Cheng Huang

Supplementary Table S1. Number of triads in CEN and GIN

Triad	frequency	fold	z-score	expectation
		CEN		
T_1	954,949	1.66	113.92	573,496
T_2	1,826,449	2.21	326.39	825,488
T_3	0	0.00	-781.93	1,191,842
T_4	0	0.00	-115.83	190,572
		GIN		
T_1	8,733	4.39	94.28	1,988
T_2	117,785	0.86	-23.84	137,299
T_3	36,744	1.28	15.72	28,604
T_4	224,283	1.02	3.44	219,654

* The z-score was calculated by random sampling 1,000 times. Fold is the ratio of observed number of triads compared to the average number of random triads.
