# Supplementary material for: Link Clustering Reveals Structural Characteristics and Biological Contexts in Signed Molecular Networks
Source: PLoS One. 2013 Jun 24;8(6):e67089. doi: 10.1371/journal.pone.0067089 (PMC3691148; doi:10.1371/journal.pone.0067089)
Supplement: Table S2 — The top twenty enriched functions of the largest module in the CEN. (PDF) [file pone.0067089.s005.pdf]

Link clustering reveals structural characteristics and biological contexts in signed molecular networks

Chen-Ching Lin, Chia-Hsien Lee, Chiou-Shann Fuh, Hsueh-Fen Juan, Hsuan-Cheng Huang

Supplementary Table S2. The top twenty enriched functions of the largest module in the CEN.

GOID	Description	Coverage	Adj. p-value
42254	ribosome biogenesis	28.52%	2.02E-05
6364	rRNA processing	22.81%	4.04E-05
16072	rRNA metabolic process	23.19%	6.06E-05
22613	ribonucleoprotein complex biogenesis	28.52%	8.08E-05
34470	ncRNA processing	24.33%	8.48E-22
34660	ncRNA metabolic process	25.10%	8.51E-20
462	maturation of SSU-rRNA from tricistronic rRNA transcript (SSU-rRNA, 5.8S rRNA, LSU-rRNA)	10.65%	1.45E-15
42274	ribosomal small subunit biogenesis	11.79%	7.27E-15
30490	maturation of SSU-rRNA	10.65%	1.22E-14
6396	RNA processing	24.33%	2.87E-12
42273	ribosomal large subunit biogenesis	8.37%	3.91E-11
42255	ribosome assembly	6.46%	1.58E-09
16070	RNA metabolic process	28.14%	8.08E-08
70925	organelle assembly	6.46%	3.58E-07
460	maturation of 5.8S rRNA	6.08%	4.27E-07
466	maturation of 5.8S rRNA from tricistronic rRNA transcript (SSU-rRNA, 5.8S rRNA, LSU-rRNA)	6.08%	4.27E-07
447	endonucleolytic cleavage in ITS1 to separate SSU-rRNA from 5.8S rRNA and LSU-rRNA from tricistronic rRNA transcript (SSU-rRNA, 5.8S rRNA, LSU-rRNA)	4.18%	2.68E-06
478	endonucleolytic cleavage involved in rRNA processing	4.18%	3.50E-06
479	endonucleolytic cleavage of tricistronic rRNA transcript (SSU-rRNA, 5.8S rRNA, LSU-rRNA)	4.18%	3.50E-06
469	cleavage involved in rRNA processing	4.94%	1.08E-05
