# Supplementary material for: Link Clustering Reveals Structural Characteristics and Biological Contexts in Signed Molecular Networks
Source: PLoS One. 2013 Jun 24;8(6):e67089. doi: 10.1371/journal.pone.0067089 (PMC3691148; doi:10.1371/journal.pone.0067089)
Supplement: Table S3 — The top twenty enriched functions of the 2nd largest module in the CEN. (PDF) [file pone.0067089.s006.pdf]

Link clustering reveals structural characteristics and biological contexts in signed molecular networks

Chen-Ching Lin, Chia-Hsien Lee, Chiou-Shann Fuh, Hsueh-Fen Juan, Hsuan-Cheng Huang

Supplementary Table S3 – The top twenty enriched functions of the 2nd largest module in the CEN.

GOID	Description	Coverage	Adj p-value
6099	tricarboxylic acid cycle	8.98%	4.07E-05
46356	acetyl-CoA catabolic process	8.98%	4.07E-05
9109	coenzyme catabolic process	8.98%	4.07E-05
51187	cofactor catabolic process	8.98%	6.11E-05
45333	cellular respiration	15.10%	8.15E-05
6084	acetyl-CoA metabolic process	9.80%	1.02E-04
9060	aerobic respiration	13.06%	1.43E-04
6732	coenzyme metabolic process	12.24%	2.04E-04
42773	ATP synthesis coupled electron transport	5.31%	2.85E-04
42775	mitochondrial ATP synthesis coupled electron transport	5.31%	2.85E-04
6119	oxidative phosphorylation	5.31%	3.05E-04
22904	respiratory electron transport chain	5.31%	3.26E-04
15986	ATP synthesis coupled proton transport	4.49%	3.67E-04
15985	energy coupled proton transport, down electrochemical gradient	4.49%	3.67E-04
32787	monocarboxylic acid metabolic process	8.57%	3.87E-04
6097	glyoxylate cycle	2.45%	4.07E-04
15992	proton transport	4.49%	4.28E-04
9206	purine ribonucleoside triphosphate biosynthetic process	4.90%	4.89E-04
9145	purine nucleoside triphosphate biosynthetic process	4.90%	4.89E-04
46487	glyoxylate metabolic process	2.45%	5.09E-04
