# Supplementary material for: Link Clustering Reveals Structural Characteristics and Biological Contexts in Signed Molecular Networks
Source: PLoS One. 2013 Jun 24;8(6):e67089. doi: 10.1371/journal.pone.0067089 (PMC3691148; doi:10.1371/journal.pone.0067089)
Supplement: Text S1 — Supplementary methods. (PDF) [file pone.0067089.s007.pdf]

Link clustering reveals structural characteristics and biological contexts in signed molecular networks

Chen-Ching Lin, Chia-Hsien Lee, Chiou-Shann Fuh, Hsueh-Fen Juan, Hsuan-Cheng Huang

# Supplementary Methods

## Biological relationships

In our analysis, six biological relationships between genes—co-regulation, PPI, within/between protein complex, shared protein domain, and duplicated genes—were used to discover the embedded biological contexts of biological links. The yeast transcription network was obtained from a previous study[1], which contained 13,385 regulations among 4,503 genes (158 transcription factors and 4,369 target genes). Two genes that were regulated by one transcription factor were defined as a co-regulated gene pair. The co-regulation relationship was only applied to investigate the embedded biological contexts of coexpressions. The other five biological relationships were used on GI analysis. Yeast PPI data were obtained from DIP (Database of Interacting Proteins)[2], which contained experimentally validated physical interactions between yeast proteins. A total of 25,198 PPIs between 4,859 proteins were collected. Information regarding the yeast protein complex was downloaded from CYC2008 at http://wodaklab.org/cyc2008/ (release 2.0, November 2010)[3]. Herein, we collected 408 yeast protein complexes covering 1,627 proteins. Complex relationships between genes defined here were determined by whether the paired proteins participated in at least one common protein complex. In contrast, protein pairs with no shared protein complex were defined as "between complex". The protein domain information was downloaded from Superfamily v1.75[4]. A total of 3,668 proteins and 2,213 domains were collected. Protein pairs that were assigned to at least one common domain were defined as co-domain. Gene duplication information was inferred from whole genome duplicated (WGD) gene pairs that were retrieved from a previous study[5]. In total, 450 duplicated gene-pairs between 900 genes were used in this study.

## Partition Density

The definition of partition density was introduced by Ahn *et al.*[6]. For a network $G$ with $E$ edges and $N$ nodes, $\{C_1, C_2, C_3, \dots, C_k\}$ is a partition of the edges into $k$ communities. Community $C_k$ has $E_k$ edges and $N_k$ connected nodes. Then, we can define the partition density of $C_k$ as $D_k = \frac{E_k-(N_k-1)}{N_k(N_k-1)/2-(N_k-1)}$. Finally, $D_G$, which is the partition density of $G$, is the average of $D_k$ weighted by the proportion of present edges and defined as $D_G = \frac{2}{E}\sum_k E_k \frac{E_k-(N_k-1)}{(N_k-1)(N_k-2)}$.

## Functional enrichment analysis

The potential functions of communities were predicted as significantly over-represented gene ontology (GO)[7] biological processes of member genes. A hypergeometric test was used to determine which GO terms were significantly over-represented ($P \leq 0.01$) in communities. A hypergeometric test was performed to determine whether the GO terms were significantly over-represented. The hypergeometric distribution is described as:

$$P(X = k) = \frac{\binom{m}{k}\binom{N-m}{n-k}}{\binom{N}{n}}$$

where $X$ denotes the evaluated functional category in GO; $N$ represents the number of GO-annotated genes of yeast; $n$ represents the number of yeast genes that are annotated as the evaluated GO functional category; and $m$ represents the number of GO-annotated genes participating in the tested communities. Thus, this formula calculates the probability of the evaluated functional category covering $k$ genes in communities. The calculated $P$-value was then adjusted by applying the Benjamini and Hochberg multiple-testing procedures to control the false-discovery rate (FDR)[8]. Finally, the functions of communities were summarized from the enriched functions manually.

## Random networks and triad evaluation

To evaluate the significance of the number of each triad ($K_3$) type, 1,000 random networks were constructed with the label-shuffling strategy. Each random network is copied from the original network, but the signs of links were randomly shuffled. Therefore, the whole network structure would be kept. The *z*-score was calculated according to the below equation.

$$\mathrm{z} = \frac{\left(K_{3,o} - \overline{K_{3,r}}\right)}{\sigma\left(K_{3,r}\right)}$$

where $K_{3,o}$ is the observed number of one $K_3$ type and $K_{3,r}$ is the random value. $\overline{K_{3,r}}$ and $\sigma\left(K_{3,r}\right)$ represent the average number and standard deviation of $K_3$ of 1,000 random networks, respectively. The fold is the ratio of observed number of $K_3$ compared to the average number of $K_3$ of 1,000 random networks.

## References


1 Jothi, R. *et al.* Genomic analysis reveals a tight link between transcription factor dynamics and regulatory network architecture. *Mol. Syst. Biol.* **5**, 294, (2009).

2 Salwinski, L. *et al.* The Database of Interacting Proteins: 2004 update. *Nucleic Acids Res.* **32**, D449-451, (2004).

3 Pu, S., Wong, J., Turner, B., Cho, E. & Wodak, S. J. Up-to-date catalogues of yeast protein complexes. *Nucleic Acids Res.* **37**, 825-831, (2009).

4 Gough, J., Karplus, K., Hughey, R. & Chothia, C. Assignment of homology to genome sequences using a library of hidden Markov models that represent all proteins of known structure. *J. Mol. Biol.* **313**, 903-919, (2001).

5 Kellis, M., Birren, B. W. & Lander, E. S. Proof and evolutionary analysis of ancient genome duplication in the yeast Saccharomyces cerevisiae. *Nature* **428**, 617-624, (2004).

6 Ahn, Y. Y., Bagrow, J. P. & Lehmann, S. Link communities reveal multiscale complexity in networks. *Nature* **466**, 761-764, (2010).

7 Ashburner, M. *et al.* Gene ontology: tool for the unification of biology. The Gene Ontology Consortium. *Nature Genet.* **25**, 25-29, (2000).

8 Benjamini, Y. & Yekutieli, D. The control of the false discovery rate in multiple testing under dependency. *Ann. Stat.* **29**, 1165-1188 (2001).
